# An Advanced Histologic Method for Evaluation of Intestinal Adenomas in Mice Using Digital Slides

**DOI:** 10.1371/journal.pone.0151463

**Published:** 2016-03-14

**Authors:** Jennifer S. Davis, Vineet Gupta, Mihai Gagea, Xiangwei Wu

**Affiliations:** 1 Department of Clinical Cancer Prevention, Unit 1013, The University of Texas MD Anderson Cancer Center, Houston, TX, United States of America; 2 Department of Veterinary Medicine and Surgery, Unit 0063, The University of Texas MD Anderson Cancer Center, Houston, TX, United States of America; Georgetown University, UNITED STATES

## Abstract

**Background and Methods:**

Mice are used for modelling the biology of many human diseases, including colorectal cancer (CRC). Mouse models recapitulate many aspects of human disease and are invaluable tools for studying the biology, treatment and prevention of CRC. Unlike humans, many mouse models develop lesions primarily in the small intestine, which necessitates removal and examination of this organ in order to evaluate treatment efficacy. Commonly, the small intestine is visually examined for gross lesions and then selectively embedded in paraffin blocks for further microscopic analysis. Unfortunately, this method suffers from inherent bias toward counting large lesions and simultaneously missing smaller lesions. Even more, this method leaves no permanent record of diagnosed and measured lesions. We evaluated inter-observer variability in a mouse model of CRC using visual examination, and directly compared the visual, gross examination with a histologic analytic method using digital slides of hematoxylin and eosin stained tissue sections.

**Results:**

Using visual examination, there was a high degree of inter-observer variability. As this method does not provide a permanent record of measurements, there is no capability to arbitrate between differing observations. In contrast, histologic analysis allowed for the creation of a permanent record of lesion measurements taken. When compared directly, histologic analysis of annotated digital images has significantly improved accuracy. Using this method we were able to distinguish mutant mice from wild type littermates even at a very young age. With gross visual examination, this distinction was not possible.

**Conclusion:**

Histologic analysis of digital images of murine intestinal tissue provides a vital improvement over the commonly used visual, gross examination method. Unlike visual gross examination, histologic analysis is not biased by the size of intestinal adenoma, misdiagnosis of another lesion type, or presence of a Peyer’s patch. It also provides accountability in the form of a permanent record of lesions counted. Histologic analysis using digital slides represents a critical improvement over the current, widely used method of visual gross examination and should be considered for future studies using mouse models of CRC.

## Introduction

Mouse models are extensively used to evaluate therapeutic interventions for intestinal adenomas as a surrogate for colorectal cancer (CRC) prevention and treatment. Mice with a mutation or deletion in the *Apc* (adenomatous polyposis coli) gene are widely used to mimic both inherited and sporadic forms of CRC. The first such mouse model was the *Apc*^*Min/+*^ mouse, which harbors a premature stop codon in one allele of the *Apc* gene [1,2]. This model primarily develops adenomas in the small intestine and only rarely in the colon. Because of this, the development and regression of lesions is not readily evaluable by colonoscopy or endoscopy, necessitating removal and direct examination of the intestinal tract.

Commonly, the intestinal tract mucosa is examined grossly for visibly raised areas and other visible lesions. This may be done with or without a contrast agent, such as methylene blue. Though rapid and widely used, this method is subject to a number of limitations. First, it is biased toward counting mostly large adenomas and missing microscopic adenomas. Small intestinal adenomas, which may not be raised in relation to the surrounding epithelium, are likely to be missed. This is of particular concern for experiments performed in younger mice, which have almost exclusively small adenomas. Second, grossly visible raised areas of intestinal mucosa may be mistakenly identified as adenomas. Normal tissue structures such as Peyer’s patches, which are normal lymphoid tissue present just beneath the epithelium of the small intestine [3] may be misidentified as adenomas, especially when there is an activation or hyperplasia of lymphoid tissue. Intestinal lesions caused by other diseases may also be misdiagnosed grossly as intestinal adenomas. Third, these inaccuracies lead to inherent variability in reading and data interpretation between observers. Gross visual exam does not provide a permanent record of the lesions counted or measurements taken, which makes it impossible to arbitrate and corroborate the results between observers with differing counts. Here, we report a digital histologic method for quantifying intestinal adenomas that addresses all three major concerns of traditional visual gross examination.

## Materials and Methods

### Mice

This study was carried out in strict accordance with the recommendations in the Guide for the Care and Use of Laboratory Animals of the National Institutes of Health. All animal experiments were reviewed and approved by the MD Anderson Animal Care and Use Committee. Mice carrying the Apc^Min/+^ genotype on the C57Bl6 background were originally obtained from Jackson Laboratories (JAX) and a breeding colony was established. For these experiments, the animals were housed in a modified barrier facility and maintained on standard chow ad libitum. Following euthanasia, intestinal tissue was harvested, flushed with PBS and either rolled into a Swiss roll in a tissue cassette, or split longitudinally and flattened on a piece of filter paper.

### Tissue Processing

Small intestinal tissues (either in the cassette or on filter paper) were fixed for 48 hours in 10% neutral buffered formalin. Tissues in the Swiss roll preparation were taken directly through tissue-processing and embedding in paraffin blocks, while tissues attached to filter paper were removed to PBS for 24 hours and then the nodular lesions of the intestinal mucosa were counted grossly on a dissecting microscope (Nikon SMZ800) at 2x magnification. Adenomas were counted in two categories, nodules greater than 2mm and nodules less than or equal to 2mm diameter. To identify smaller lesions, a clean plastic bacterial loop was run across the tissue to detect small raised areas. After adenomas were counted grossly on the dissecting microscope, each length of intestine was then rolled up and placed into a tissue cassette that was immersed into 10% neutral buffered formalin and submitted to tissue processing and paraffin embedding followed by microtome cutting into 5μm–thick sections. Hematoxylin and eosin (H&E) staining was performed on serial sections at every 350μm interval through the tissue, which resulted in 12–14 stained slides per intestine of each animal.

### Slide Processing and Adenoma Counting

Stained slides were scanned using an Aperio ScanScope XT slide imager and digital image files were created. Images were analyzed at 200x magnification and observed lesions were annotated using Aperio ImageScope software (v.12.0.5039). Adenomas and hyperplasias were annotated and digital snapshots, saved as jpg pictures, were captured for each slide and lesion. Digital images of serial sections from the same intestine were compared side by side to ensure that lesions larger than 400μm were not counted more than once. For adenomas that were visible on multiple slides, measurements were taken on each slide, but were annotated as the same recurring lesion. Adenoma size was measured at the maximum width (measured parallel to the longitudinal plan of the intestine); adenomas appearing in more than one slide were categorized according to the maximum width measured. All measurements were exported into a separate spreadsheet for each animal. A veterinary pathologist (Dr. Gagea) reviewed four slides with sections at approximately 1mm intervals from each of five mice in a blinded fashion. The results of counted and annotated adenomas were then unblinded and compared to the investigators counts. The slides were then reviewed jointly by Drs. Gagea and Davis for clarity, lesions matching and agreement. The initial count, final agreement and change in adenoma number are reported.

### Statistical Analysis

Where noted, adenoma counts are reported as average with standard deviation. Student’s *t* test was used to analyze the significant differences between groups.

## Results

**Fig 1:**
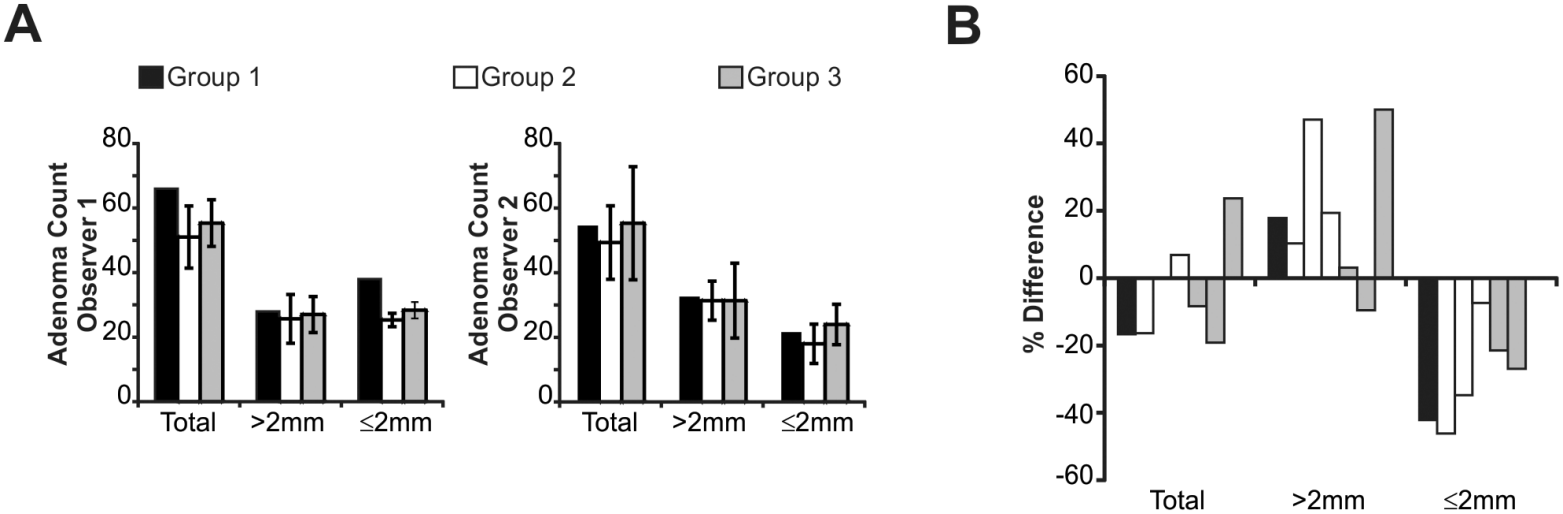
Lesion counting is inconsistent between observers. (A) Adenomas were counted by two observers, blind to the mouse group as well as the lesion count of the other observer. Columns represent the average count. n = 3 per group, except Group 1 where n = 1, Bars = Stdev. (B) Percent difference between observers by animal, using the same animals as in (A) Each column represents a single animal.

To assess inter-observer variability, intestinal lesions were counted independently by two observers using gross visual examination in a blinded fashion. There was a high degree of variability between observers in both the overall (Fig 1A) and individual animal counts (Fig 1B).

**Fig 2:**
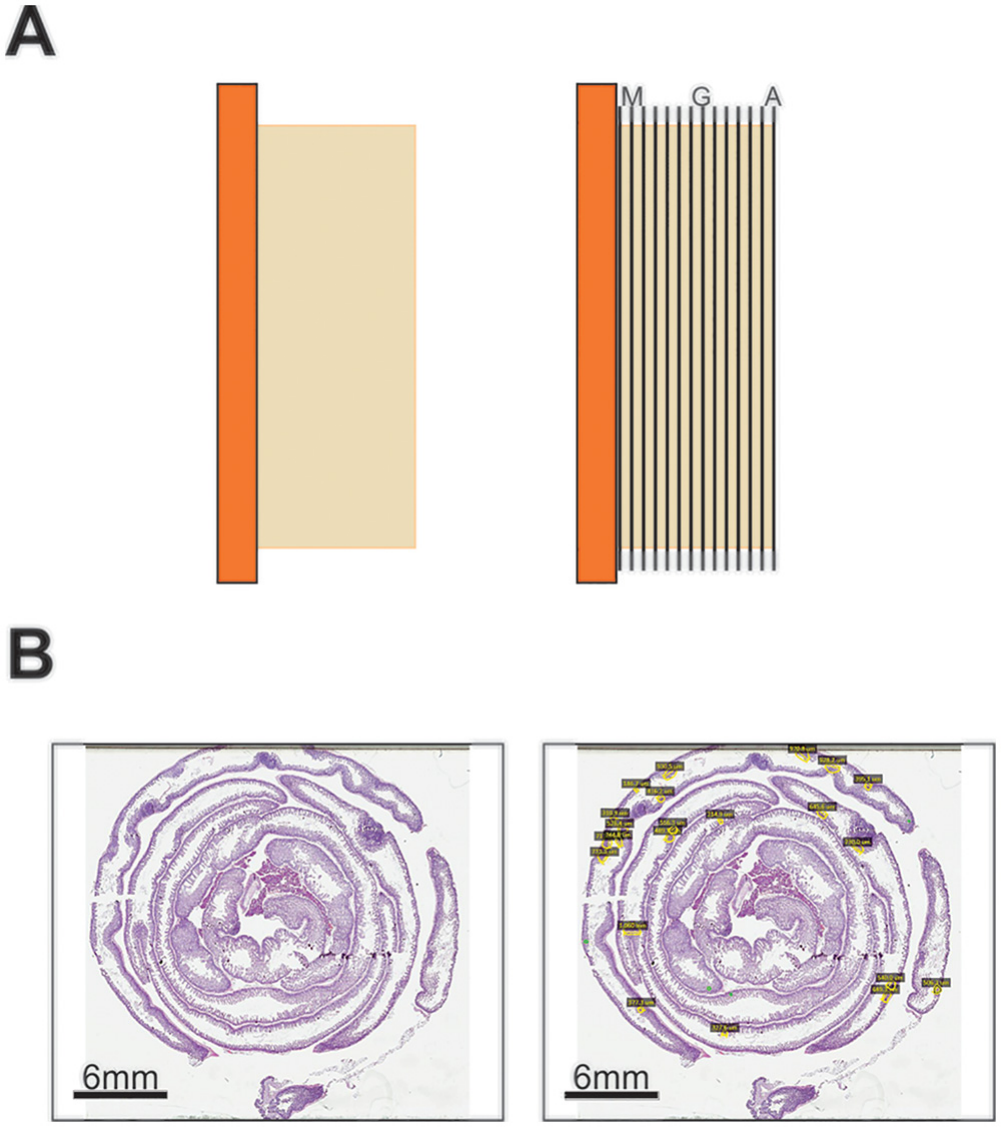
Illustration of methods. (A) Side-view diagrams of paraffin block (left) and approximate position of stained sections, where ‘A’ indicates first section, ‘G’ indicates seventh section and ‘M’ indicates thirteenth section (right). (B) Digital snapshots of whole slides before (left), and after (right) lesion annotation.

**Fig 3:**
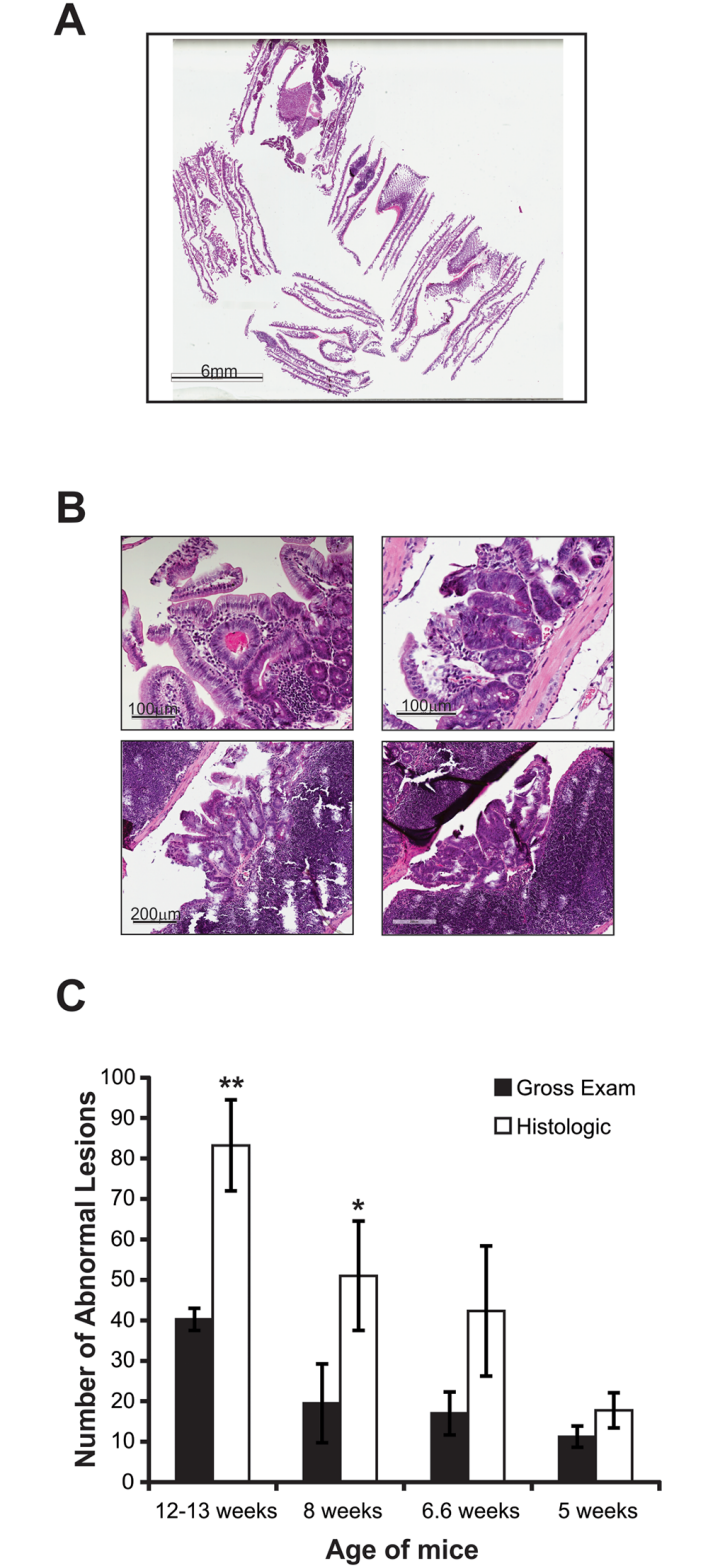
Histologic evaluation of intestinal tissue. (A) Snapshot image of an entire digital slide. (B) Intestinal lesions identified on the slide depicted in (A) Upper left: representative hyperplasia of mucosal epithelium that could be miscounted as adenoma on visual gross exam. Upper right: small adenoma that could be easily missed at visual gross exam. Lower left: adenoma adjacent to Peyer’s patch on the first serial section from slide shown in (A) Lower right: the same adenoma from lower left image appearing on the next serial section cut approximately 350μm deeper into the paraffin block. (C) Average lesions per age group. For tissue counted histologically, lesion number includes hyperplasias and adenomas. Columns represent the average lesion count. Bars = standard deviation. n = 4 mice per age group, except 6.6 weeks old, where n = 3. * p < 0.01 comparing total lesions, ** p <0.05 comparing adenomas only, p < 0.001 comparing total lesions using Student’s t-test.

Following the advice of a veterinary pathologist, we began examining adenomas microscopically, using digital slides. This was accomplished by placing intestinal tissues into modified Swiss roll formation [4], processing and embedding tissues in paraffin blocks, and performing H&E stained serial sections through the tissues at every 350μm (Fig 2A). Stained slides were then scanned and evaluated (Fig 2B). We then performed a direct comparison of the two methods. Mice were sacrificed at various ages; intestines were examined grossly for visual counting of adenomas and then were embedded for histologic exam as above (Fig 3A). Tissues were analyzed in a blinded fashion and lesion counts were compared between gross visual and histologic exam (Table 1). Notably, visual examination identified 24 large adenomas (>2mm diameter), compared to 1 adenoma of this size identified using histologic evaluation (Table 1). Since tissues can shrink up to 20% during processing into paraffin blocks [5], the largest adenomas were re-classified as >1.6mm. Reclassification increased the number of large adenomas to 4 on histologic exam compared to 24 on visual exam (Table 1). This discrepancy is likely due to the misclassification of Peyer’s patches (visible in Fig 3B lower images) as large adenomas.

**Table 1:** Lesion Count by Age.

	Gross Visual Count (Histologic Count)			
Age	Total	>2mm (>2mm, >1.6mm)	≤2mm	Hyperplasias
**12–13 weeks**				
Mouse 1	43 (41)	2 (0,0)	41 (41)	27
Mouse 2	37 (51)	1 (0,2)	36 (51)	35
Mouse 3	42 (53)	3 (1,1)	39 (52)	42
Mouse 4	39 (49)	3 (0,1)	36 (49)	35
**8 weeks**				
Mouse 1	29 (35)	3 (0,0)	26 (35)	19
Mouse 2	17 (21)	1 (0,0)	16 (21)	15
Mouse 3	7 (28)	2 (0,0)	5 (28)	18
Mouse 4	25 (44)	0 (0,0)	25 (44)	24
**6.6 weeks**				
Mouse 1	23 (35)	3 (0,0)	20 (35)	19
Mouse 2	15 (34)	3 (0,0)	12 (34)	15
Mouse 3	13 (11)	1 (0,0)	12 (11)	13
**5 weeks**				
Mouse 1	11 (13)	0 (0,0)	11 (13)	11
Mouse 2	15 (7)	0 (0,0)	15 (7)	7
Mouse 3	10 (9)	2 (0,0)	8 (9)	7
Mouse 4	7 (0-WT)	0 (0-WT)	7 (0-WT)	0-WT
Mouse 5	9 (7)	0 (0,0)	9 (7)	10

Unlike visual examination, histologic analysis enables the identification of small adenomas (≤0.5mm) and hyperplastic lesions of the mucosal epithelium that would otherwise go undetected. When plotted as intestinal lesions (adenomas plus hyperplasias), the difference between the counting methods increases with age (Fig 3C).

In the youngest group of mice, one wild type (WT) mouse was included to test the sensitivity of both methods for distinguishing WT from *Apc*^*Min/+*^. Adenomas were counted in every animal under gross visual exam; however, one mouse had no identifiable abnormal lesions (adenomas or hyperplasias) when assessed histologically. Un-blinding the data revealed that this animal was WT (Table 1).

Because this method requires that ability to distinguish adenomas from normal tissue structures under microscopic examination, a veterinary pathologist reviewed four slides from each of five selected mice. The pathologist was initially blinded to the adenoma counts of the investigator/observer. The counts were then compared and slides were jointly reviewed for agreement. The initial count, final count and change in count are shown in Table 2. The total counts for each mouse were not adjusted (Table 1) as only select mice and slides were subjected to this additional review.

**Table 2:** Adenoma Counts Before and After Pathologist Review.

Age	Original Count	Count After Joint Review	Change in Adenoma Count
**8 weeks**			
Mouse 2			
Slide ‘B’	4	5	+1
Slide ‘E’	1	1	0
Slide ‘H’	2	2	0
Slide ‘K’	3	3	0
Mouse 3			
Slide ‘B’	2	1	-1
Slide ‘E’	2	3	+1
Slide ‘H’	3	4	+1
Slide ‘K’	4	3	-1
Mouse 4			
Slide ‘B’	5	4	-1
Slide ‘E’	6	5	-1
Slide ‘H’	6	7	+1
Slide ‘K’	5	5	0
**5 weeks**			
Mouse 1			
Slide ‘B’	0	0	0
Slide ‘E’	2	2	0
Slide ‘H’	2	1	-1
Slide ‘K’	2	2	0
Mouse 3			
Slide ‘B’	1	1	0
Slide ‘E’	1	1	0
Slide ‘H’	0	0	0
Slide ‘K’	2	2	0

## Discussion

The ubiquitous use of mouse models for investigating the pathogenesis and treatment of CRC supports the need for accurate and consistent evaluation of intestinal adenomas. In this study, we demonstrate that there is a high degree of variability between individual observers using visual gross evaluation of the same tissue (Fig 1), which may lead to inconsistency between experiments and a lack of reproducibility between investigators. This discrepancy can be addressed by the routine use of a histologic method as proposed here.

Previously, other investigators have used a similar histologic method to verify adenomas in a limited number of mice, reporting the difference in large adenomas observed per slide [6]. The method used did not take into account two facts: adenomas are not distributed evenly throughout the intestine, and large adenomas will appear in multiple histological sections. By contrast, evaluation of the entire intestine using the new histologic counting method and digital images that we have described, gives a more accurate and complete picture of the state of carcinogenesis in each animal.

One drawback of the new histologic evaluation is that this method is more time consuming than the gross visual examination. However, two important factors mitigate this. First, the significant difference in adenomas and lesions counted (Fig 3) is likely to influence the outcome of experiments. Second, the inability of gross visual examination to distinguish WT from *Apc*^*Min/+*^ mice at the youngest age examined is critical when considering experiments with young mice (Table 1). Therefore, although more time consuming, use of this method addresses both, diagnosis accuracy problems arising from gross visual examination methods and significant improvement in minimizing the inter-observer counting variation (Table 1 and Fig 3). Finally, the new histologic evaluation of intestinal tissues using digital slides creates a permanent record of measurements taken, and allows the data to be re-examined by other investigators who may then agree or disagree with the initial diagnosis, providing accountability and accuracy, as demonstrated by our additional pathologist’s review (Table 2). Importantly, the intestinal adenoma counts by the pathologist were within ±1 adenoma per slide on each of the slides reviewed from 5 animals (see Table 2), and the net change in the final adenoma count was also within ±1 adenoma per animal.

## Conclusions

Accurate and reproducible animal studies must be conducted with accountability in order to translate reliably the relevant animal scientific discoveries into the clinic as soon as possible. The new histologic analysis method described here provides significantly better accuracy, reproducibility and accountability, and should be considered in the analysis of future studies using mouse models of CRC.
